# The Pattern of Respiratory Disease Morbidity and Mortality in a Tertiary Hospital in Southern-Eastern Nigeria

**DOI:** 10.1155/2013/581973

**Published:** 2013-12-17

**Authors:** Victor Aniedi Umoh, Akaninyene Otu, Henry Okpa, Emmanuel Effa

**Affiliations:** ^1^Department of Internal Medicine, University of Uyo, Uyo, Akwa Ibom State, Nigeria; ^2^University of Calabar, Nigeria; ^3^Department of Internal Medicine, University of Calabar, Nigeria

## Abstract

*Background*. Respiratory complaints are commonly encountered in medicine and respiratory diseases place a high burden on healthcare infrastructure. Healthcare planning should be based on adequate information: this study will help us to analyze the pattern of respiratory disease admissions in the medical wards in a developing country. *Methods*. The medical records of patients admitted into the medical wards over a 5-year period were retrieved and reviewed. Information obtained included demography, diagnosis, comorbid conditions, and risk factors for respiratory disease. *Results*. Three thousand four hundred and ninety patients were admitted into the medical wards with 325 (9.3%) of them diagnosed with a respiratory condition. There were 121 females and 204 males. The average age of the patients was 40.7 ± 14.7 years. Only 7% of the patients smoked cigarette. The commonest respiratory conditions were tuberculosis (66.8%) and pneumonia (24.9%). The commonest comorbidity was HIV infection (39.7%). Tuberculosis/HIV coinfection rate was 50.7%. HIV infection was the single most important predictor of an adverse outcome (OR 5.1, 95% CI 2.05–12.7, *P* < 0.001). *Conclusion*. Infective conditions make up a large percentage of respiratory diseases in low income countries with HIV infection constituting a significant risk factor for a poor disease outcome.

## 1. Introduction

Respiratory complaints such as cough and catarrh are some of the commonest symptoms encountered in medicine. This is due in part to the large surface area; nearly 70 m^2^ of the lungs present to the atmosphere [1]. The atmosphere that we breathe is more than just “air.” In reality, it is a complex mixture of ambient gases and environmental particulates to which pathogen containing droplets are added when respiratory secretions are coughed or sneezed out by others. Respiratory diseases constitute a major cause of morbidity and mortality worldwide. The top four respiratory diseases, lower respiratory tract infections, chronic obstructive pulmonary disease (COPD), tuberculosis, and lung cancer, are among the ten leading causes of death worldwide [2]. In Africa, lower respiratory tract infection and tuberculosis are ranked 2nd and 8th, respectively [3].

In developed countries, respiratory diseases feature prominently in the top ten causes of morbidity and mortality [4–7]. In Nigeria, lower respiratory tract infections constituted the second leading cause of death in all age brackets in 2002, a year in which TB was the seventh leading cause of death, accounting for 4% of all deaths [8]. In India another developing country, pneumonia and pulmonary tuberculosis ranked in the top five causes of death [9].

Health care demands are rapidly expanding and the trends are changing in developing countries. Upgrading of the health care system is a pressing need and the priorities are not always easy to define especially in a resource constrained system. The changes necessary to improve the health care systems should be evidence-based. There are few studies on the morbidity and mortality pattern of respiratory diseases in Africa and fewer still from Nigeria. This study was undertaken to address some of the issues concerning respiratory diseases in Nigeria.

## 2. Methods

This was a retrospective study conducted in the University of Calabar Teaching Hospital (UCTH), Calabar Cross River state in the South-Eastern part of Nigeria. This Hospital is a 400-bed hospital with 40 beds spaces for medical admissions. The hospital serves as a training centre for undergraduate medical students and postgraduate Resident Doctors. It is also a referral centre for other secondary health facilities in this part of Nigeria.

The medical records of all patients admitted into the medical wards of UCTH over a five-year period from April 2008 to March 2013 were retrieved and reviewed and the following information was extracted: demographic data, diagnosis of respiratory disease, comorbidities, complications, duration of hospitalisation, cigarette smoking habit, and treatment outcome. Treatment outcome was defined as favourable if the patient was successfully treated and discharged or unfavourable if the patient died while on admission or discharges against medical advice (DAMA). Data was analysed using the computer program Statistical Package for the Social Sciences, version 16.0 (SPSS Inc., Chicago, IL, USA). Descriptive and frequency statistics were obtained for the variables studied. The chi-square test was used to evaluate associations between the categorical variables, and values of *P* < 0.05 were considered statistically significant.

## 3. Results

### 3.1. General Characteristics of the Patients

Three thousand four hundred and ninety patients were admitted into the medical wards within the period of study. There were 2202 males and 1288 females with a male to female ratio of 1.7 : 1. Three hundred and twenty five patients were diagnosed with respiratory diseases constituting 9.3% of all the medical admissions. Two hundred and four were males (9.3% of all male patients) and one hundred and twenty one were females (9.4% of all female patients). The average age for the males was 41.4 ± 14.3 years with a range of 15–85 years, while for the females it was 39.3 ± 15.2 years with a range of 19–85 years. Sixty-four percent of the patients were less than 45 years old and 92% were less than 65 years old (Table 1). Most of the patients were farmers with <5% being unemployed. Smoking was not common among the patients with only 7% smoking rate and only the males indulged in smoking.

### 3.2. Respiratory Diagnosis

**Figure 1:**
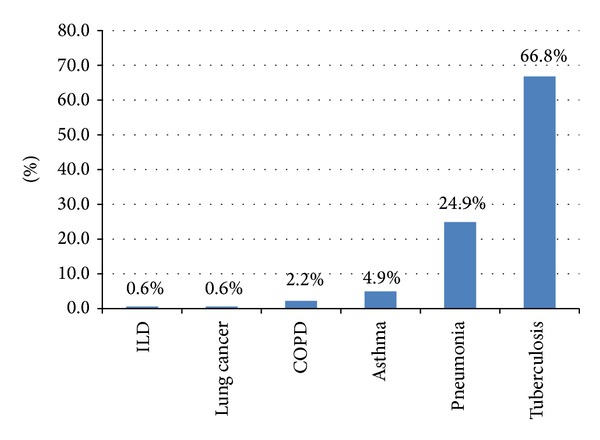
Frequency of respiratory conditions among patients in the medical wards.

Figure 1 shows the frequency of respiratory diseases among the patients. 217 (66.8%) patients were diagnosed with pulmonary tuberculosis, 81 (24.9%) with pneumonia, 16 (4.9%) patients with asthma, 7 (2.2%) patients with COPD, and only 2 (0.6%) patients with lung cancer and interstitial lung disease. The distribution of the various conditions among the patients is shown in Table 2. There was no significant gender difference in frequency of chest disease. The age distribution of the chest diseases shows that tuberculosis and pneumonia were more common within the 30–44 years age bracket and least common within the 65+ age bracket. Asthma and COPD were more common within the 45–64 years age bracket followed by the 15–29 and 30–44 years age bracket. There was also one case of interstitial lung disease (ILD) in the age brackets of 30–44 and 65+. There was one case of lung cancer among patients aged 34–44 years and 45–64 years. The differences were statistically significant (*P* = 0.001). Smoking was significantly associated with COPD, lung cancer, and chest infections (*P* = 0.007). HIV infection was significantly associated with tuberculosis and pneumonia (*P* < 0.0001).

### 3.3. Comorbid Conditions

**Figure 2:**
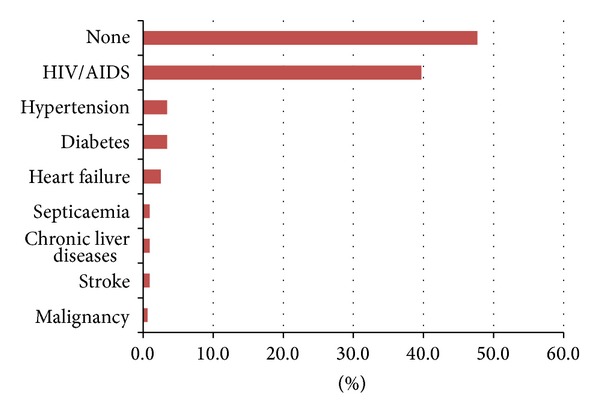
Frequency of comorbidities among respiratory diseases patients.

Figure 2 shows the distribution of comorbidities among the patients with chest diseases. One hundred and fifty six (48%) of the patients had one comorbidity or the other. The commonest comorbidity was HIV infection (39.7%). Eleven (3.4%) of the patients had diabetes mellitus. Eight (2.5%) of the patients had congestive cardiac failure. There were three patients each with stroke, chronic liver disease, and septicaemia, while two patients had other malignancies other than lung cancer.

### 3.4. Duration of Hospitalisation

The average duration of hospitalisation was 15.4 ± 6.4 days with a range between 3 and 35 days. Patients with interstitial lung disease spent the most days in hospital (21 ± 9.9) followed by patients with tuberculosis and lung cancer, 17.6 ± 5.2 days and 17 ± 11.3 days, respectively. Asthma patients spent the least number of days in hospital; 8.3 ± 2.5 days (Table 3). A one way analysis of variance test was conducted to explore the impact of the respiratory diagnosis on the duration of hospital stay. Patients were divided into six groups based on their diagnosis. There was a statistically significant difference in duration of hospital admission for the six groups *F*(5,319) = 38.9, *P* < 0.0001. The diagnosis had a large effect on the duration of hospitalisation (Eta squared 0.37). Post hoc analysis using Tukey-HSD indicated that the duration of stay for tuberculosis was significantly longer than that of asthma and pneumonia.

### 3.5. Treatment Outcome

**Figure 3:**
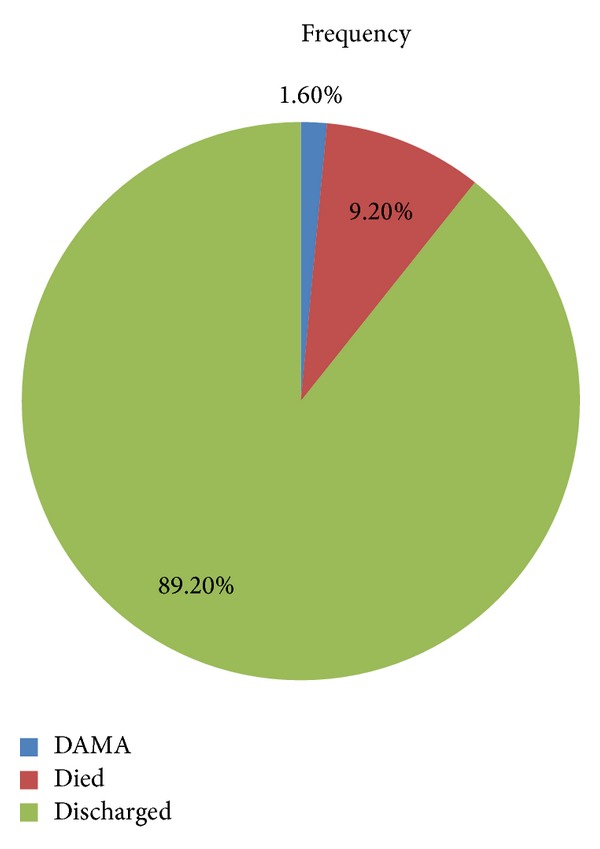
Outcome among patients with respiratory disease.

Two hundred and ninety (89.2%) patients were treated successfully and discharged form hospital and 30 (9.2%) died while on admission, while five (1.6%) patients DAMA; Figure 3. All 35 (10.8%) patients had an unfavourable outcome. There was no significant association between the diagnosis and the final outcome (Table 4).

Direct logistic regression analysis was performed to assess the impact of certain factors on an unfavourable outcome. The model contained seven variables: age, duration of hospital stay, gender, cigarette smoking, HIV infection, tuberculosis, and lung cancer. The full model was statistically significant *χ* ^2^ = 29.066 and *P* < 0.001, indicating that the model could distinguish patients who had a favourable outcome from those with an unfavourable outcome. The model could explain up to 17.3% of the variance in outcome (Negelkerke *R* squared) and correctly classified 90% of the patients. Only HIV infection made a unique significant contribution to the model (OR 5.1, *P* < 0.0001) indicating that patients with HIV infection were over five times more likely to have an unfavourable outcome (Table 5).

## 4. Discussion

Respiratory diseases constituted 9.3% of medical admissions in this survey. Similar observations have been reported by other investigators. Desalu et al. [10] in Ilorin, North-Central Nigeria, reported that respiratory diseases make up 8.7% of all medical admissions. Respiratory diseases accounted for a higher percentage (14.5%) of medical admissions in Saudi Arabia [11] and 31.73% in Kathmandu, Nepal [12]. The much higher prevalence of respiratory diseases in the Saudi survey and Kathmandu may be due to the higher prevalence of a major risk factor for respiratory diseases in these desert areas with many pilgrims: cigarette smoking which may be as high as 53% in some of these areas [13, 14] among other factors. This stands in contrast to the observed smoking rate in this study (7%) as well as in a previous Nigerian report of 8.7% [15].

Tuberculosis was the commonest respiratory condition followed by pneumonia. Together they account for over 90% of respiratory admissions. In Ilorin Central Nigeria, chest infections were the most important causes of respiratory disease hospitalisation with tuberculosis and pneumonia occupying the first and third most frequent indications for hospitalisation [10]. This contrasts sharply with observations from other parts of the world where chronic noninfective respiratory diseases predominate [11, 12].

Young patients <45 years made up >60% of the admissions in this study. This picture is similar to what was observed in a previous study from Nigeria by Desalu et al. [10] in that study most of the patients were young adults <45 years. This can be explained by the demographic pattern of Nigeria where >80% of the population is <45 years [16]. This pattern contrasts with observations in the ward admissions of patients with respiratory diseases in other parts of the world where older patients make up the bulk of the respiratory diseases admissions [4–6, 11].

The most common comorbidity in this study was HIV infection with a prevalence of 39.7% and a tuberculosis coinfection rate of 50.7%. HIV has been shown to have a strong association with tuberculosis. Pennap et al. [17] in Keffi, North-Central Nigeria reported a prevalence of 44.2% for tuberculosis/HIV coinfection. Studies from regions with a low prevalence of HIV have reported a different pattern for the comorbidities; diabetes and hypertension being the most common comorbidities [11].

The average duration of hospitalisation was 15.4 ± 6.4 days. A previous local study reported the duration of hospitalisation for medical admissions of 15.6 ± 13.8 days [18]; this shows that on average respiratory disease patients did not spend more time on admission than medical conditions of other systems. Hospital stay in this study was significantly longer for ILD and tuberculosis patients compared with pneumonia and asthma (*P* < 0.05). This is similar to observations by Desalu et al. [10] where they reported the average duration of hospitalisation for respiratory diseases to be 14 days with pneumonia and asthma accounting for the shortest duration of hospitalisation. Alamoudi [11] in Saudi Arabia reported similar findings; a majority of asthma and pneumonia patients were hospitalised for less than a week, while ILD, tuberculosis, and bronchiectasis accounted for most of the patients that spent more than two weeks on admission.

Thirty (9.2%) patients died while five patients DAMA. Lung cancer recorded 100% mortality in this study. Mortality from lung cancer is high all over the world [19]. Smoking is a major risk factor for lung cancer [20]. In this study, all the patients with lung cancer had a significant smoking history. The smoking rate is on the increase in low income countries [3] as such we should expect an increase in the incidence and mortality from lung cancer in the coming years. In this study one death was attributed to COPD. With the increasing rate of smoking in low income countries we should also expect an increase in morbidity and mortality from this condition.

11.5% of patients with tuberculosis had an unfavourable outcome. Different investigators have reported varying mortality rates for tuberculosis. Our observations on mortality is similar to a report of mortality rate of 10.1% among 4000 tuberculosis patients in Ethiopia by Tessema et al. [21] and Busari et al. [22] in Ido-Ekiti South-West, Nigeria. A much higher mortality rate was observed by Salako and Sholeye in Sagamu, South-West, Nigeria. In their study 23.2% of the hospitalised patient had unfavourable outcome [23]. The different mortality rates may be a reflection of the difference in diseases severity in those centres.

HIV infection was the only condition with a unique significant contribution to an adverse outcome in this study after controlling for confounders (OR 5.1, 95% CI 2.05–12.7, and *P* < 0.001). The probability of dying or abandoning treatment among patients with respiratory disease was five times higher for patients with concomitant HIV infection than for patients without HIV infection. Several studies from Nigeria have described the critical role of HIV infection as a cause of morbidity and mortality in hospitalised patients [24, 25]. Studies have also revealed that HIV/AIDS patients in Nigeria tend to present late with advanced illnesses [26]. Thus compounding the outcome in this very serious disease condition.

There are several limitations of this study. First of all this was a retrospective study as such we encountered some poor and incomplete record keeping which affected our ability to quantify some variables. It is worthy to note that certain diseases, such as bronchiectasis, pulmonary vascular diseases, sleep apnoea, pneumocystis jirovecii pneumonia (PCP), sarcoidosis, collagen lung diseases, overlap syndromes of asthma and COPD, and pneumoconiosis were not reported. This may be due to a lack of awareness and a low index of suspicion from the attending physicians. In conclusion this study has highlighted the high burden of respiratory diseases in our hospital and the role of tuberculosis and HIV coinfections as a major cause of morbidity and mortality.

## Figures and Tables

**Table 1:** General characteristics of patients with respiratory diseases in UCTH.

	Female 121 (%)	Male 204 (%)	Total 325 (%)
Age			
15–29	41 (33.8)	42 (20.6)	83 (25.5)
30–44	38 (31.5)	87 (42.7)	125 (38.5)
45–64	33 (27.3)	58 (28.4)	91 (28)
65+	9 (7.4)	17 (8.3)	26 (8)
Occupation			
Farmer	42 (34.7)	78 (38.2)	120 (37.0)
Trader	15 (12.4)	35 (17.1)	50 (15.4)
Public servant	31 (25.6)	52 (25.5)	83 (25.5)
Semiskilled	23 (19.0)	34 (17.7)	57 (17.5)
Unemployed	10 (8.3)	5 (2.5)	15 (4.6)
Smoker	0 (0.0)	23 (11.3)	23 (7.1)

**Table 2:** Distribution of respiratory conditions among patients.

	Tuberculosis *N* = 217 (%)	Pneumonia *N* = 81 (%)	Asthma *N* = 16 (%)	COPD *N* = 7 (%)	Lung ca. *N* = 2 (%)	ILD *N* = 2 (%)	*P*
Gender							
Female	80 (36.9)	28 (34.6)	9 (56.2)	3 (42.9)	0 (0.0)	1 (50.0)	0.53
Male	137 (63.1)	53 (65.4)	7 (43.8)	4 (57.1)	2 (100)	1 (50.0)	
Age							
15–29	60 (27.6)	18 (22.2)	4 (25)	1 (14.3)	0 (0.0)	0 (0.0)	0.001
30–44	90 (41.5)	29 (35.8)	4 (25)	0 (0.0)	1 (50.0)	1 (50)	
45–64	58 (26.7)	20 (24.7)	7 (43.8)	5 (71.4)	1 (50.0)	0 (0.0)	
65+	9 (4.2)	14 (17.3)	1 (6.2)	1 (14.3)	0 (0.0)	1 (50)	
HIV+ve	110 (50.7)	18 (22.2)	1 (6.2)	0 (0.0)	0 (0.0)	0 (0.0)	<0.001
Smoking	17 (7.8)	5 (6.2)	0 (0.0)	2 (28.6)	2 (100)	0 (0.0)	0.007

Lung ca.: lung cancer

ILD: interstitial lung disease.

**Table 3:** Average duration of hospital stay based on the respiratory condition.

Diagnosis	Mean (SD)	95% CI	
		Lower	Upper

Asthma	8.25 (2.5)	6.91	9.59
Pneumonia	9.14 (5.0)	8.03	10.25
Tuberculosis	17.63 (5.2)	16.94	18.33
Lung cancer	17.0 (11.3)	−84.65	118.65
COPD	16.14 (5.7)	10.85	21.44
ILD	21.0 (9.89)	−67.94	109.94

ILD: interstitial lung disease.

**Table 4:** Diseases outcome according to respiratory condition.

	Asthma 16 (%)	Pneumonia 81 (%)	Tuberculosis 217 (%)	Lung ca. 2 (%)	COPD 7 (%)	ILD 2 (%)	*P*
Discharged	16 (100)	74 (91.4)	192 (88.5)	0 (0.0)	6 (85.7)	2 (100)	0.147
DAMA	0 (0.0)	1 (1.2)	4 (1.8)	0 (0.0)	0 (0.0)	0 (0.0)	
Died	0 (0.0)	6 (7.4)	21 (9.7)	2 (100)	1 (14.3)	0 (0.0)	

Lung ca.: lung cancer

ILD: interstitial lung disease.

**Table 5:** Predictors of unfavourable outcome among patients with respiratory conditions.

Parameter	OR	*P*	95% CI for OR	
			Lower	Upper
Age	0.99	0.96	0.97	1.03
Hospital stay	0.98	0.58	0.91	1.05
Gender	0.5	0.09	0.22	1.13
Smoking	2.74	0.17	0.65	11.5
HIV infection	5.1	<0.001	2.05	12.7
TB infection	1.03	0.94	0.36	2.97
Lung cancer	2.01 × 10^10^	0.99	0.00	
